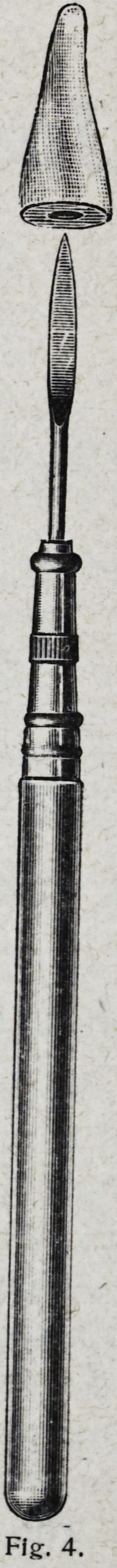# A Simple Means of Increasing the Security of Post Crowns

**Published:** 1907-10

**Authors:** T. C. Trigger

**Affiliations:** St. Thomas, Ont., Can.


### A SIMPLE MEANS OF INCREASING THE SECURITY OF POST CROWNS.

BY T. C. TRIGGER, L. D. S., D. D. S., St. Thomas, Ont., Can.

There are various methods used to increase the retention of post crowns so as to prevent their dislodgment by the severe strain to which they are subjected, and to overcome any displacement whatever, the utmost rigidity of the pin is necessary, and also the contiguous substance surrounding the pin requires an equal resisting strength. To accomplish this to a considerable degree, I have adopted a means of increasing the security of crown posts, which I have used for a number of years with success.

The technique of this method is as follows: After preparing the root in the usual manner to receive a post crown by using suitable facers for paring the end of the root beneath the gum margin. The next operation is to prepare the root canal by selecting an Ottolengui reamer the desired size as is required for the case at hand, Fig. 1. The result a uniform cut of the canal will be obtained. After having this done make a cone of about 32 gauge plate gold so that

Fig. 3.

Fig. 2.

Fig. 1.

Fig. 4.

it will easily pass into the already prepared root, this cone is very easily made by forming it around a tapered instrument, the edges slightly overlapping as in Fig. 2. The upper part of the cone should be cut off so as to admit the post to pass through.

The length of this cone should be less than the depth of which the root has been reamed, this will allow the piece of gold to pass easily in place when setting the crown. Fig. 3 shows the root prepared to receive the metal cone. Before inserting the cone in the root before the final insertion of the crown, mix a sufficient quantity of cement of a creamy consistency, and apply it to the extreme end of the root with a minature spatula, Fig. 4. While the cone is forced partly in position, and additional cement is applied therein, this amount will entirely fill the root canal. Now the crown post is carried into the tube in the final forcing of the crown in place, the cone at the same time is driven with it, causing a binding effect on the walls of the cavity and also results in the cone closing in against the post, thereby increasing the security of the crown.

When the crown and this attachment are permanently in place the end of the cone rests against the base of the crown, thereby increasing the rigidity of the crown, and also protects the post from being broken in this situation, which is so liable to occur in post crowns where no strength is given to the metal that forms the post.

## Figures and Tables

**Fig. 1. f1:**
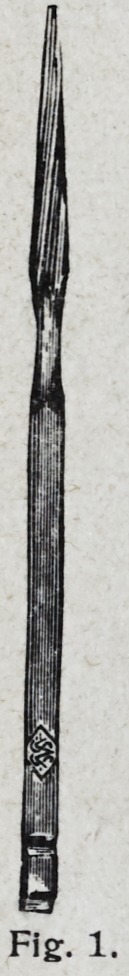


**Fig. 3. f2:**
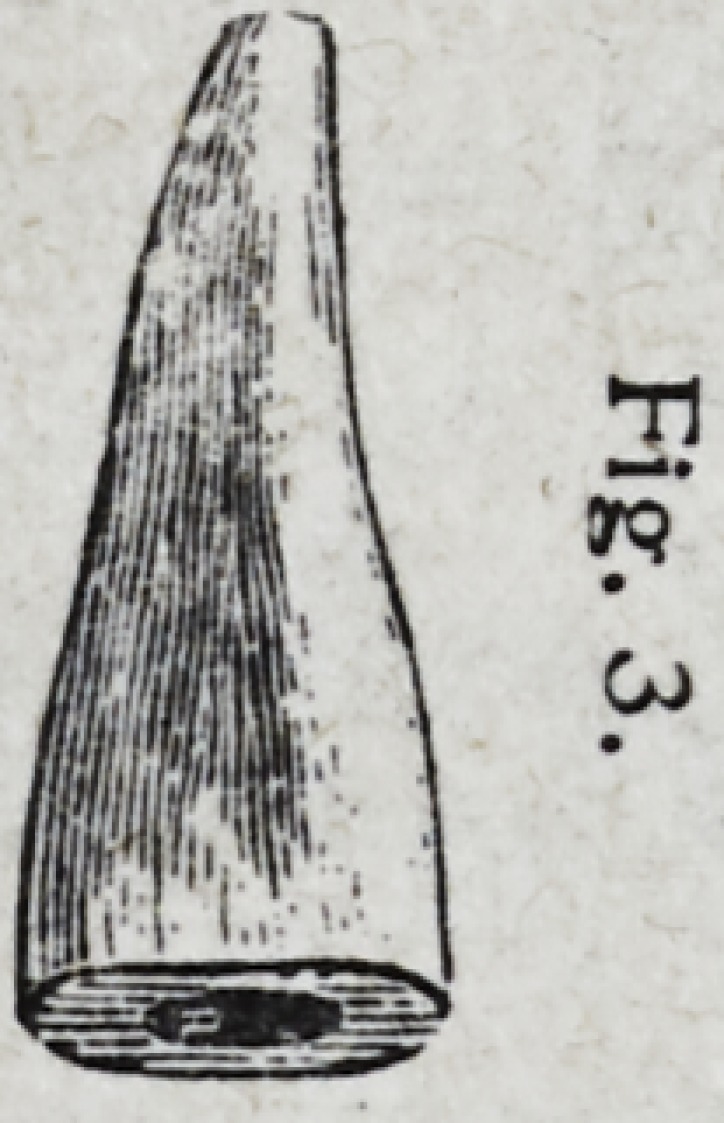


**Fig. 2. f3:**
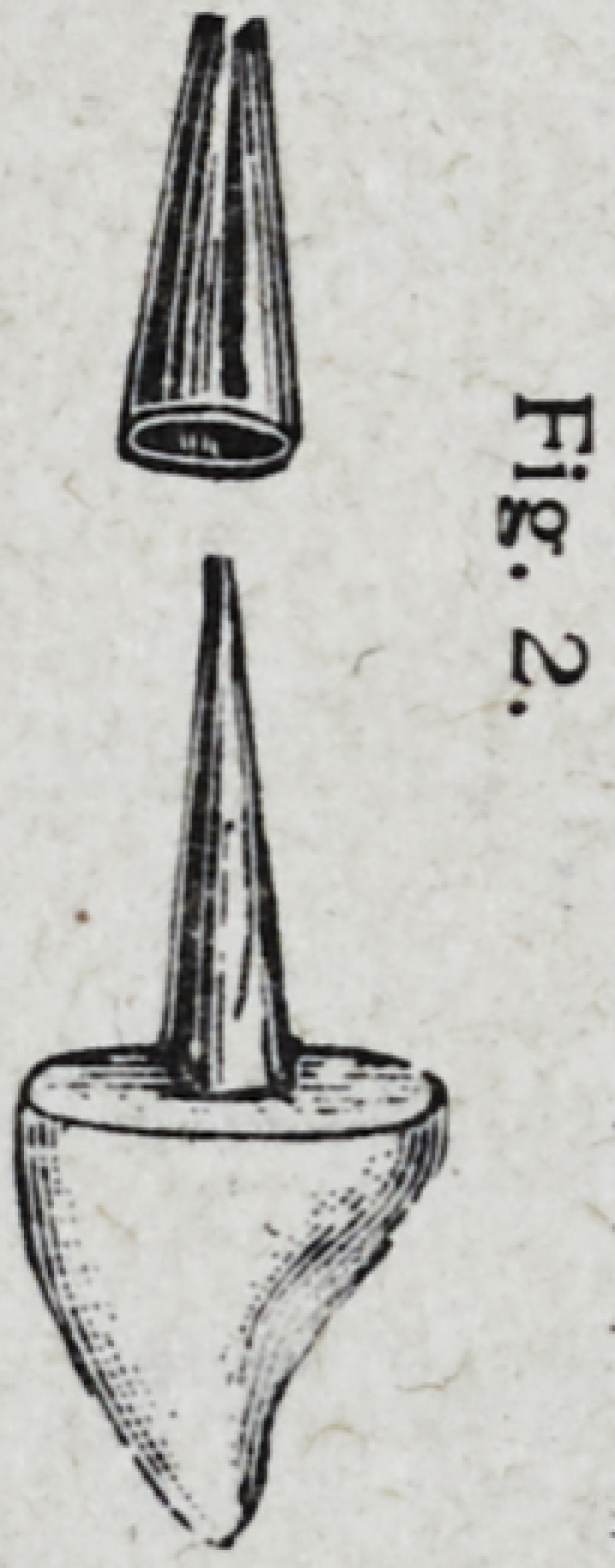


**Fig. 4. f4:**